# CircRNA SOD2 motivates non-small cell lungs cancer advancement with EMT via acting as microRNA-2355-5p’s competing endogenous RNA to mediate calmodulin regulated spectrin associated proteins-2

**DOI:** 10.1080/21655979.2021.2024331

**Published:** 2022-02-19

**Authors:** Changsheng Lv, Yiying Hu, Xin Zhou, Yuntao Zhu, Jin Wang, Fachen Zhou

**Affiliations:** a Department of Thoracic Surgery, The First Affiliated Hospital of Dalian Medical University, Dalian City, Liaoning Province, China; b Department of Neuroelectrophysiology, The First Affiliated Hospital of Dalian Medical University, Dalian City, Liaoning Province, China; c Department of Histology and Embryology, Dalian Medical University, Dalian City, Liaoning Province, China

**Keywords:** Circular RNA SOD2, MicroRNA-2355-5p, CAMSAP2, non-small cell lung cancer, invasion and migration, epithelial-mesenchymal transition

## Abstract

Circular RNAs (circRNAs) are closely linked with human cancer development such as non-small-cell lung cancer (NSCLC). However, the characteristics and specific functions of most circRNAs in NSCLC remained unknown. Previous studies have suggested that circRNA SOD2 (CircSOD2) expression was upregulated in a number of cancers. This study aimed to explore the functions of circSOD2 in NSCLC advancement with epithelial-mesenchymal transition (EMT). Expression profile analysis of circSOD2, miR-2355-5p, and calmodulin-regulated spectrin-associated protein 2 (CAMSAP2) was detected by real-time quantitative PCR (RT-qPCR). Transwell assay, cell migration assay, CCK8, ELISA, RIP assay, RNA pull-down assay, and Western blot analysis were performed to evaluate the functions of circSOD2, miR-2355-5p, and CAMSAP2. We found elevated expression of circSOD2 and CAMSAP2 while reduced expression of miR-2355-5p in NSCLC tumor tissues. Silencing or overexpression of CircSOD2 resulted in increased or decreased expression of miR-2355-5p, respectively. Mechanically, we showed that silencing of CircSOD2 and overexpression of miR-2355-5p resulted in the reduced rate of NSCLC cell proliferation. Inhibition of miR-2355-5p reversed the changes induced via silencing of CircSOD2. MiR-2355-5p binds to the CircSOD2 promoter and triggered its stimulation, which further activated circSOD2 expression. CircSOD2 suppression impaired lung cancer cell growth, cell migration, prohibited cell cycle progression, and in vivo tumor growth by targeting miR-2355-5p expression in NSCLC tissues. Meanwhile, increased expression of CAMSAP2 reversed the changes stimulated by the elevated level of miR-2355-5p in NSCLC progression. This innovative signaling axis CircSOD2/miR-2355-5p/CAMSAP2 illustrated the new horizon to investigate NSCLC tumorigenesis and provided new prognosis and treatment of NSCLC.

## Graphical abstract

**Figure:**
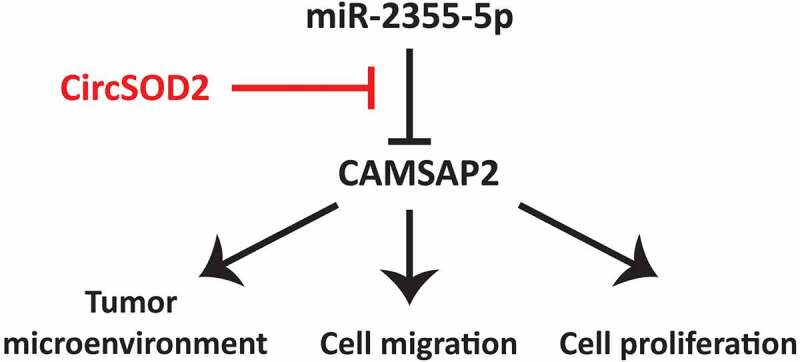

## 1 Introduction

Cancer is the major reason of global morbidity and mortality [1]. According to an estimate, by the year 2030, there will be 22 million cases of cancer along with 13 million cancer-related deaths [1]. Lung cancer (LC) is a heterogeneous disorder with elevated incidence and metastatic potential. LC is clinically classified into two categories, small-cell LC (SCL) and non-small-cell lung cancer (NSCLC). Among them, NSCLC is the most extensively diagnosed type of cance, and is supposed to combine with lung adenocarcinoma (LUAD), lung squamous cell carcinoma (LUSC), and large cell LC (LULC) [2]. Regarding cure strategies, the first-line defense for NSCLC can be, such as platinum-based dual chemotherapy, the targeted therapy and surgical resection. However, the clinical prognostic data of NSCLC are not sufficient. A study has clarified that the 5-year survival rate of NSCLC patients is just 18% [3]. Lack of specific and precise therapies against NSLC makes it a potential target for further investigation to explore its mechanism and treatment strategies.

Circular RNAs (circRNAs) are a group of covalently bonded noncoding RNA (ncRNA), which are more stable than linear RNA [4]. Recently, a large number of researchers have found a close relationship between circRNAs and cancer and focus on their role as miRNA sponges and therefore their effect in the cancer-related pathways [5]. Lung cancer contributes about 11.8% of all cancer diagnosed cancer [6]. A study has clarified that circRNAs are available to be applied as biomarkers for NSCLC diagnosis and prognosis [7]. Functionally, circRNAs have also been investigated to modulate a series of malignant phenotypes of NSCLC cells [8,9]. It has been suggested that circRNAs have the potential to work together with microRNAs (miRs) to control their targeted genes and prevent miRNA from interacting with mRNA in the 3′ untranslated region. Therefore work as competitive endogenous RNA (ceRNA) to ultimately impact the NSCLC advancement [10]. On the other hand, CircSOD2 is regarded as an oncogene of hepatocellular carcinoma (HCC), results in an increased HCC cell proliferation rate, and leads to tumorigenesis *in vivo* [11].

Studies have demonstrated that increased expression of CircSOD2 leads to increased liver cancer cell growth and is associated with cancer progression in vivo. It established that the elevated level of CircSOD2 inhibited the miR-502-5p expression, and moreover, it encourages DNMT3a expression and stimulates the JAK2/STAT3 signaling pathway [11]. In another in vivostudy, CircSOD2 was associated with neointima SMCs in balloon-injured rat carotid arteries. Prominently, silencing of CircSOD2 decreased the injury-activated neointima development by reducing the neointimal SMC proliferation. Consequently, CircSOD2 might be a possible target for preventing the progression of proliferative vascular diseases [12].

Because of its high stability, circRNA has been considered as a potential candidate as new biomarkers of lung cancer [13]. F-circEA is extracted from EML4-ALK fusion gene and is a common example for lung cancer circRNAs, while expression of F-circEA gene can be detected in plasma samples of lung cancer patients [14]. Furthermore, circRNAs might work as good prognostic biomarkers for treatment [15–17].

A number of studies on genome-wide RNA sequencing analysis of hepatocellular carcinoma cells and tissues have demonstrated that expression of CircSOD2-derived circRNA is higher in cancer tissues as compared to its neighboring normal liver tissues [18]. However, the role of CircSOD2 in NSCLC remains unknown. Thus, in order to clarify the role of miR-2355-5P/CricSOD2 in NSCLC, we first evaluated the expression level of miR-2355-3p and how it is associated with CircSOD2 in NSCLC. Then, the expression of miR-2355-3p as a diagnostic biomarker for NSCLC was investigated in the current piece of research, while evaluation has been performed on the basis of the expression level in NSCLC patients and healthy individual’s serum. The CircSOD2 is found as primary target gene of miR-2355-3p and would be verified. The modulatory mechanism of ceRNA among CircSOD2, miR-2355-5p. and CAMSAP2 was also investigated.

## 2 Materials and methods

### 2.1 General data

In total, 73 NSCLC cancer patients’ samples were collected from the Department of Oncology in The First Affiliated Hospital of Dalian Medical University. Among the patients, 46 were males and 27 were females with the age between 35 and 72 (60.21 ± 8.96) years. On the basis of tumor size, 73 cases were divided into two classes: one with a tumor diameter of 3 cm or less (51 cases) and the other with a tumor diameter of more than 3 cm (22 cases). On the basis of the type of cancer, it has been found that 41 cases suffered adenocarcinoma, with 32 cases of squamous cell carcinoma. Patients were further classified according to the tumor node metastasis (TNM) staging: 32 cases of stage I, 22 cases of stage II, 11 cases of stage III, and 8 cases of stage IV. There were 47 cases with lymph node metastasis, 26 cases with nonmetastasis, 7 cases of distant metastasis, and 66 cases of nonmetastasis. The degree of tumor differentiation is as follows: 11 cases in stage I, 37 cases in stage II, and 25 cases in stage III. All patients were clinically diagnosed with the cancer and had no history of chemotherapy or radiotherapy before surgical treatment. The surgically removed tissues consisted of cancerous tissue and adjacent normal tissue that was over 5 cm from the edge of the cancerous tissue. This study has been approved by the Ethics Committee of the First Affiliated Hospital of Dalian Medical University, and consent form has been signed by patients and patient’s family for tumor sampling.

### 2.2 Cell culture

Human normal bronchial epithelial (HBE) cell lines (Catalog number: PCS-300-010) and human NSCLC lines (HCC827, H1299, H1975, and A549) were obtained from American Type Culture Collection (ATCC) (VA, USA). Cells have been cultured and maintained in different kinds of growth media according to the culture type and provided with 10% Fatal bovine serum (FBS). Cell growth culture medium RMP11640, MEM, Dulbecco’s Modified Eagle Medium, and FBS were obtained from HyClone Biological Company (USA) [19].

### 2.3 Cell transfection

Lung cancer A549 and H1975 cells were seeded into 6-well plates for 80% confluency before plasmid transfection. Transfection has been performed by strictly following the instructions of the lipofectamine 2000 kit (Invitrogen, Carlsbad, CA, USA) and transfection of si-CircSOD2#1/CircSOD2#2/negative control (NC), miR-2355-5p inhibitor/mimic, inhibitor/mimic-NC, and pcDNA-CAMSAP2/NC, and the medium was changed after 6-h transfection. After 48 h of plasmid transfection, cells have been harvested for subsequent experiments. Synthesis and preparation of all sequences and plasmids were via GenePharma Co. Ltd. Company (Shanghai, China) [20].

### 2.4 Reverse transcription quantitative polymerase chain reaction (RT-qPCR)

To determine the expression of different genes at the transcriptional level, RT-qPCR has been performed, while BETrizol (Bao Biological Engineering Co., Ltd., Dalian, China) reagent has been used to extract the total RNA from tissues and cells. The RNA concentration and purity have been determined by using nanodrop. To prepare cDNA, a PrimeScript RT kit have been used, with SYBRGREEN real-time PCR Master Mix for real-time PCR reaction in ABI 7500 real-time PCR system. Application of the 2^−ΔΔCt^ method [21] was used for calculating the relative expression quantity. Experiments have been performed three times, and results are analyzed as averaged. Synthesis of the primers was via Guangzhou Qingke Biotechnology Company, and the sequences are manifested in Table 1 [19].

**Table 1.** The primer sequences

Genes	Primer sequences (5ʹ – 3ʹ)
miR-2355-5p	Forward: CTGAGGGATCCC CAGATACAATGG
U6	Forward: 5ʹ-TGCGGGTGCTCGCTTCGGCAGC
circSOD2	Forward: AAACCACGATCGTTATGCTG
	Reverse: CGTTAGGGCTGAGGTTTGTC
CAMSAP2	Forward: GCCAAAATCGCCTGCAATCTG
	Reverse: ACGACAGTATAGTTCAGCCGATA
GAPDH	Forward: AGAAGGCTGGGGCTCATTTG
	Reverse: AGGGGCCATCCACAGTCTTC

### 2.5 Western blot

To elucidate the expression of different proteins in the cells, cells A549 and H1975 have been cultured and maintained in PRMI and DMEM, respectively, for a determined period of time. After that, cells were washed with PBS and trypsinized and incubated on ice with protein lysis buffer for 30 minutes. After that, samples were centrifuged at 4°C for 10 min at 12000rpm. Supernatant has been transferred to the new Eppendorf, and the protein concentration was measured. Determination of the protein concentration was in line with the instructions of the bicinchoninic acid kit (AmyJet Scientific, Wuhan, Hubei, China). SDS-PAGE was prepared, the protein was separated by gel electrophoresis, and then electroblot of the protein was transferred onto the nitrocellulose membrane. After blocking with 5% BSA (10-L16, Beijing Zhongsheng Likang Technology Co., Ltd., China), incubation was performed with the primary antibody CAMSAP2 (1: 1000), glyceraldehyde-3-phosphate dehydrogenase (GAPDH) (1: 5000) (Cell Signaling Technology, Beverly, MA, USA), E-cadherin (1: 1000), N-cadherin (1: 1000) (Abcam, Cambridge, MA, USA), and horseradish peroxidase-labeled immunoglobulin G (IgG) (1: 1000, Wuhan Boster Company) secondary antibody. The PVDF membrane was analyzed by using chemiluminescence reaction solution (Pierce, USA). GAPDH was applied as the loading control, and analysis of the Western blot image was performed using ImageJ2x software. N = 3, and the data were averaged [4].

### 2.6 Cell counting kit (CCK)-8

Cell viability has been determined by using the CCK-8 8 (Beyotime Biotechnology, Shanghai, China) by following the manufacturer’s protocol. Cells have been cultured in a 96-well plate with the cell density of 4000cells/well and allowed to grow. After culturing for 24, 48, and 72 h, of cell culture, 10 μL of CCK-8 reagent (Sigma) was added to each well of the 96-well plate [9]. Absorbance was read at a wavelength of 450 nm in a plate reader (DU650, Beckman Coulter, CA, USA), while the cell viability graph has been plotted by using mean values.

### 2.7 Transwell

Cells have been resuspended in serum-free culture medium in the presence of Opti-MEMI (Invitrogen). Cells have been seeded in a matrigel (BD Biosciences, USA)-coated Transwell chamber (Corning, Corning, New York, USA). 5 × 10^4^ cells were seeded at a density of 500 μL/well. Cells have been seeded in a culture medium provided with 10% of FBS. After a determined time period, culture medium has been removed and the cells were gently wiped with a cotton swab on the upper chamber. Cells have been fixed with 4% paraformaldehyde and stained s with crystal violet. Cells have been observed and counted under a microscope. Matrigel coating was not applied in the migration experiment, and the rest of the steps were the same as the invasion. N = 3, and the data were averaged [19].

### 2.8 circRNA characterization

Detection of the circular construction of CircSOD2 was via ribonuclease R (RNase R), while GAPDH has been used as control. Total RNA extracted from A549 and H1975 cells was treated with 10 U RNase R (GENESEED, Guangzhou, China), and qPCR has been performed. The equal volume of the 1 × reaction buffer was applied as N C. N = 3, and the data were averaged [4].

### 2.9 RNA pull-down assay

RNA pull-down assay has been executed by using a pierce magnetic RNA protein pull-down kit (Ambion, Austin, Texas, USA). Briefly, biotin-labeled CircSOD2 plasmids (50 nM each) were transfected in vitro for 24 h. Afterward, cells were harvested, rinsed off, and incubated with M-280 streptavidin magnetic beads (Sigma). Then, two equal amounts of Streptavidin Beads were washed with 100 × volume binding buffer. Protein lysate from beads is mixed, blocked with the non-BSA and tRNA, and centrifuged. After centrifuging, keep the supernatant and discard the beads. Biotin-labeled RNA has been diluted in binding buffer at a 1:20 ratio and stored on ice. Mix biotin-labeled RNA with the cell lysate and 30 μL of 1 × loading buffer. Boil the sample to denature it. Finally, SDS-PAGE analysis was performed using ImageJ (n = 3) [9].

### 2.10 RNA-binding protein immunoprecipitation (RIP) assay

RNA-binding protein immunoprecipitation (RIP) assay was executed by using a Magna RNA RIP Kit (Merck KGaA, Darmstadt, Germany) by following the manufacturer’s protocol. A495 and H1975 cells were lysed by using RIP lysis buffer (Beyotime) and incubated with magnetic beads conjugated with Argonaute-2 (Ago2, Millipore, MA, USA) or control immunoglobulin G (IgG, Millipore) at 4°C overnight. Detachment of the complex was achieved with proteinase K (Sigma-Aldrich), and the bound RNA was identified by RT-qPCR. N = 3, and the data were averaged [11].

### 2.11 The luciferase activity assay

To investigate the effect of CircSOD2/CAMSAP2 3ʹ-UTR combining with miR-2355-5p A495 and H1975 cells, luciferase assay has been performed. Binding sites of CircSOD2/CAMSAP2 3ʹ-UTR have been combined with miR-2355-5p by using bioinformatics software. The 3ʹUTR sequence of the circSOD2 promoter region or CAMSAP2 sequence consisting of miR-2355-5p binding site was used to construct the CircSOD2/CAMSAP2 3ʹUTR wild-type (WT) plasmid (CircSOD2/CAMSAP2-WT) and CircSOD2/CAMSAP2 3ʹUTR-mutant (MUT) plasmid (CircSOD2/CAMSAP2-MUT). Briefly, the transfection of CircSOD2/CAMSAP2-WT/MUT plasmids and mimic NC and miR-2355-5p mimic has been performed by using Lipofectamine 2000 (Invitrogen) by following the manufacturer’s protocol. After 48 h of transfection, luciferase activity was measured using a dual-luciferase reporting kit (Promega) [19].

### 2.12 Statistical analysis

GraphPad Prism 7.0 (GraphPad, San Diego, CA, USA) was applied to process all experimental data. The data were statistically analyzed via SPSS 22.0 and presented as the mean ± standard deviation (SD) of three independent experiments. One-way analysis of variance (ANOVA) and Student’s t-test were applied to compare the differences among groups, and the Pearson test was used for correlation analysis. *P* < 0.05 was considered as statistically significant.

## 3. Results

### 3.1 CircSOD2 is elevated in NSCLC tissues and cells

**Figure 1.**
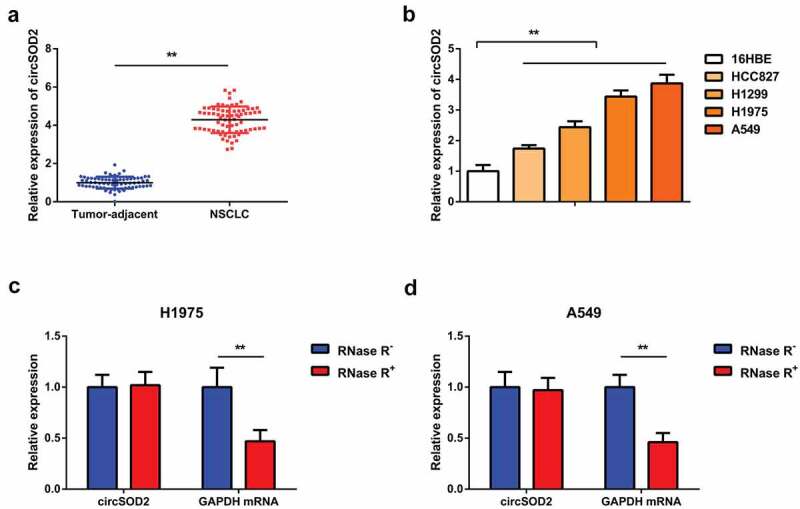
CircSOD2 is elevated in NSCLC tissues and cells. A/B: RT-qPCR to detect circSOD2 in NSCLC and corresponding adjacent tissues, and human normal bronchial epithelial cell line 16HBE and human NSCLC cell lines HCC827, H1299, H1975, and A549; C: CircSOD2 after treatment with RNase A; GAPDH was applied as control. * *P* < 0.05; **P < 0.01. N = 3. The data in the figure were manifested as mean ± SD.

A number of previous studies have found that miR-2355-3p presented an increased expression level in lung adenocarcinoma, and it can be distinguished from normal serum [22]. Hepatocellular carcinoma cells and tissues have shown elevated expression of circRNA induced by CircSOD2 in cancer tissues as compared to its neighboring normal liver tissues [18]. To date, the molecular mechanism of CircSOD2 in NSCLC needs to be addressed. The objective of our study was to investigate the whether CircSOD2 has been involved in tumorigenesis of NSCLC patients. Detection of CircSOD2 was in NSCLC and corresponding adjacent tissues, which manifested the upregulation (Figure 1a). Further detection of CircSOD2 was in 16HBE and human NSCLC cell lines. The results (Figure 1b) identified that CircSOD2 significantly increased in NSCLC cell lines, as compared to 16HBE. Among the lung cancer cell lines, A549 and H1975 cells manifested the most apparent difference, so they were selected for subsequent experiments. Additionally, it was discovered that CircSOD2 was resistant to RNase R treatment, while GAPDH was evidently digested by RNase R, emphasizing that CircSOD2 possessed a ring structure (Figure 1c). All these results assured that CircSOD2 was stably expressed and upregulated in NSCLC patients and cells.

### 3.2 CircSOD2 silencing refrains NSCLC advancement

**Figure 2.**
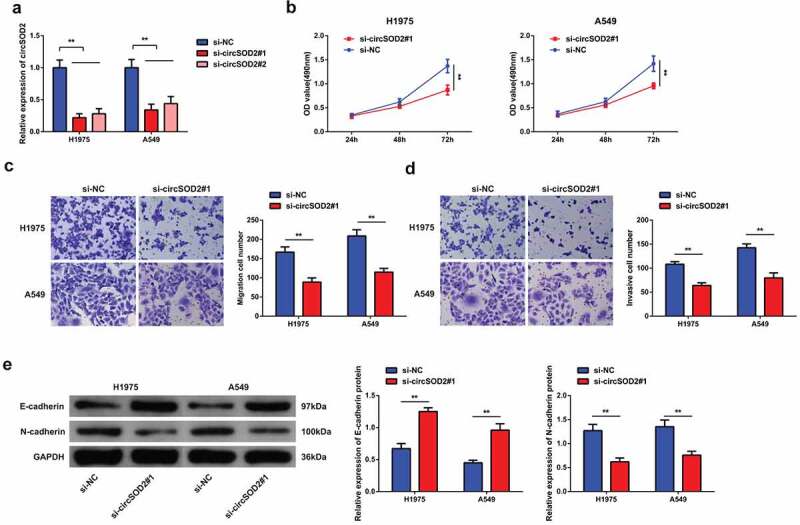
CircSOD2 silencing represses NSCLC cell advancement with EMT. A: RT-qPCR to detect circSOD2; B: CCK-8 to detect cell proliferation; C: Transwell to detect cell migration ability; D: Transwell to detect cell invasion ability; E: Western blot detection of E-cadherin and N-cadherin, after cell transfection; *P < 0.05; **P < 0.01. N = 3. The data in the figure were manifested as mean ± SD.

To investigate the effect of circSOD2 on the biological phenotype of NSCLC cells, siRNA CircSOD2 has been transfected in A549 and H1975 cells. Experimental evidences suggested that si-CircSOD2#1 and si-CircSOD2#2 could successfully knock down CircSOD2 expression in cells. On the basis of higher efficiency, si-CircSOD2#1 has been used for further transfection experiments (Figure 2a). For exploring the biological progress of A549 and H1975 cells after repressive CircSOD2, a series of experiments was conducted, manifesting the decrease in the cell proliferation by downregulating the N-cadherin expression, with increased expression of E-cadherin (Figure 2b-e). Briefly, CircSOD2 silencing repressed NSCLC cell advancement with EMT.

### 3.3 CircSOD2 performs as miR-2355-5p’s ceRNA

**Figure 3.**
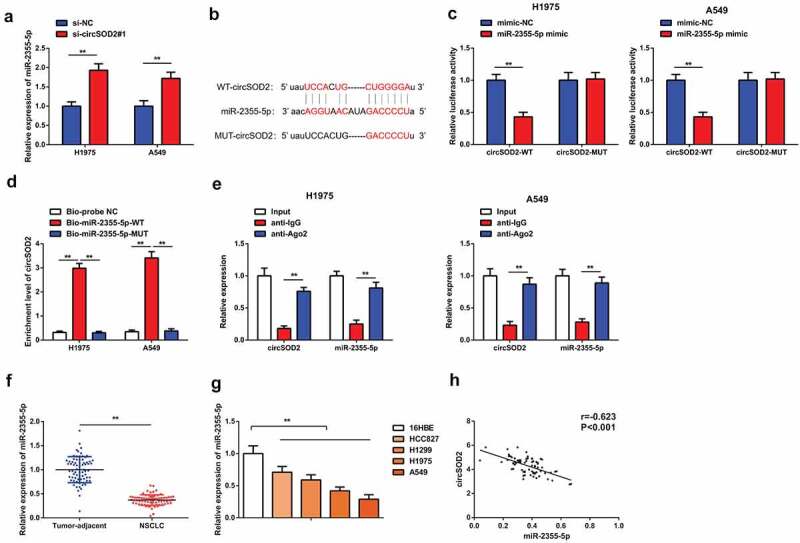
CircSOD2 performs as miR-2355-5p’s ceRNA. A: RT-qPCR to detect miR-2355-5p after cell transfection; B: Bioinformatics website starbase to predict the binding site of circSOD2 and miR-2355-5p; C: Dual luciferase verification of the binding of circSOD2 to miR-2355-5p; D: RNA pull-down to detect the enrichment of miR-2355-5p on circSOD2; E: RIP assay and qPCR to detect RNA enrichment of circSOD2 and miR-2355-5p in anti-IgG -and anti-Ago2-precipitated RNAs in cell extract of NSCLC cells; F/G: RT-qPCR to detect miR-2355-5p in NSCLC, corresponding adjacent tissues, and 16HBE and HCC827, H1299, H1975, and A549; H: Correlation analysis of circSOD2 and miR-2355-5p in NSCLC tissue; *P < 0.05; **P < 0.01. N = 3; The data in the figure were manifested as mean ± SD. The Pearson test was used to analyze the correlation.

A large number of evidences have shown that circular RNAs work as sponge and regulate the expression of miRNA [23,24]. To further explore the molecular mechanism of CircSOD2 influencing the biological phenotype of NSCLC cells, A549 and H1975 cells have been transfected with si-CircSOD2#1 plasmid and expression of miR-2355-5P has been detected. Results have shown an elevated level of miR-2355-5p after refraining CircSOD2 (Figure 3a). By searching bioinformatics website, it is found that miR-2355-5p is a target of circSOD2. The potential interaction site between CircSOD2 exon 3 and 5′ miR-502-5p is shown in Figure 3b. The luciferase activity assay verified that cotransfected miR-2355-5p mimic with the WT CircSOD2 was significantly reduced as compared to the mimic-NC, while the mutant 3ʹUTR circRNARNASOD2 remained unchanged, affirming that circSOD2 could specifically bind to miR-2355-5p, as manifested in Figure 3c. Additionally, the enrichment of CircSOD2 in the Bio-miR-2355-5p-WT was elevated steadily, while that in the Bio-miR-2355-5p-MUT manifested no changes where the Bio-probe NC is used as control (Figure 3d). Moreover, CircSOD2 and miR-2355-5p were concurrently enriched in anti-Ago2-immunoprecipitated RNAs in A549 and H1975 cells (Figure 3e). The above results emphasized that CircSOD2 modulated miR-2355-5p via competitively combining with miR-2355-5p. We also demonstrated that miR-2355-5p was downregulated in human NSCLC tumor tissues and cells, and CircSOD2 and miR-2355-5p in NSCLC tissues were negatively correlated (r = −0.621, *P* < 0.001) (Figure 3f-h).

### 3.4 Repressive miR-2355-5p turned around the changes induced via refrained CircSOD2 on NSCLC advancement

**Figure 4.**
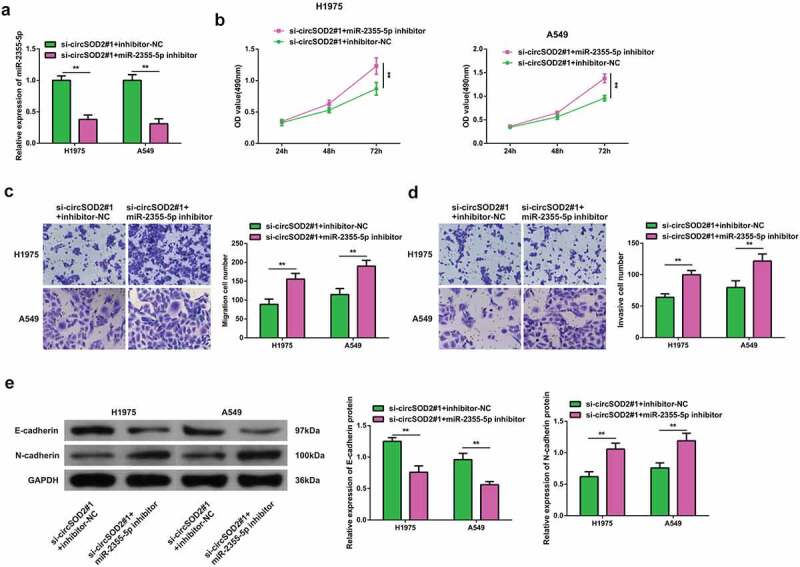
Repressive miR-2355-5p turns around the changes induced via refrained circSOD2 on NSCLC advancement. A: RT-qPCR to detect miR-2355-5p; B: CCK-8 to detect cell proliferation; C: Transwell to detect cell migration ability; D: Transwell to detect cell invasion ability; E: Western blot detection of E-cadherin and N-cadherin after cell transfection; *P < 0.05; **P < 0.01. N = 3. The data in the figure were manifested as mean ± SD.

Moreover, to elucidate if CircSOD2 has a modulatory effect on the progression of NSCLC cells by regulating the miR-2355-5p expression. In order to prove that CircSOD2 controlled the progression of NSCLC cells via modulating miR-2355-5p, A549 and H1975 cells were transfected with si-circRNARNASOD2 and miR-2355-5p inhibitor. Results have shown that miR-2355-5p inhibitor transfection successfully reversed the effects of si-CircSOD2 on miR-2355-5p (Figure 4a). A series of experiments manifested reduced miR-2355-5p and reversed the influences of si-CircSOD2 on NSCLC cell development with EMT (Figure 4b-e). These findings suggested that CircSOD2 knockdown repressed NSCLC cell advancement with EMT via sponging miR-2355-5p.

### 3.5 CAMSAP2 is the immediate target of miR-2355-5p

**Figure 5.**
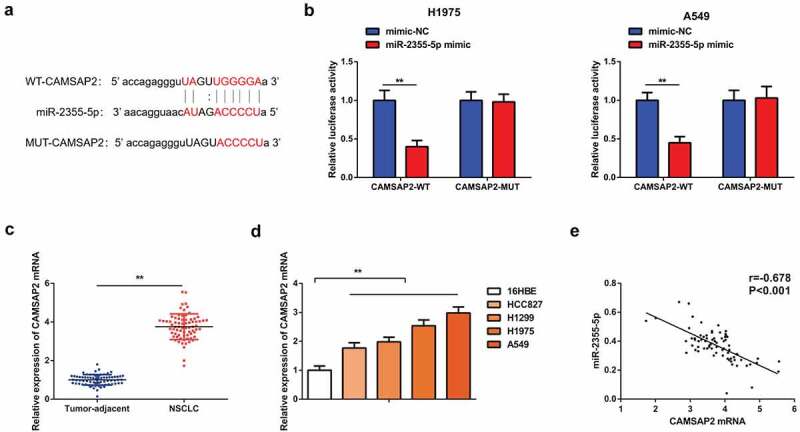
CAMSAP2 is the immediate target of miR-2355-5p. A: Bioinformatics website starbase to predict the binding site of CAMSAP2 and miR-2355-5p; B: Dual luciferase verification of the targeting of CAMSAP2 with miR-2355-5p; C/D: RT-qPCR to detect CAMSAP2 in NSCLC and corresponding adjacent tissues, in 16HBE and HCC827, H1299, H1975, and A549; E: Correlation analysis of miR-2355-5p and CAMSAP2 in NSCLC tissue; *P < 0.05; **P < 0.01. N = 3; The data in the figure were manifested as mean ± SD. The Pearson test was used to analyze the correlation.

In order to further clarify the mechanism by which the CircSOD2/miR-2355-5p axis influenced progression of NSCLC cells, targeting sites of miR-2355-5p and CAMSAP2 have been forecasted by using bioinformatics website (Figure 5a). Results show that targeting miR-2355-5p with CAMSAP2 shows that the luciferase activity of the cells cotransfected with WT CAMSAP2 and miR-2355-5p mimic was reduced, while that with MUT CAMSAP2 was not changed clearly as compared with the mimic NC, assuring that miR-2355-5p was available to target CAMSAP2 (Figure 5b). It was also discovered that CAMSAP2 was enhancive in human NSCLC tumors tissue and cells, and CAMSAP2 and miR-2355-5p in NSCLC tissues were negatively correlated (r = −0.678, *P* < 0.001) (Figure 5c-e).

### 3.6 Elevated CAMSAP2 reverses the effects of strengthening miR-2355-5p on NSCLC cell advancement

**Figure 6.**
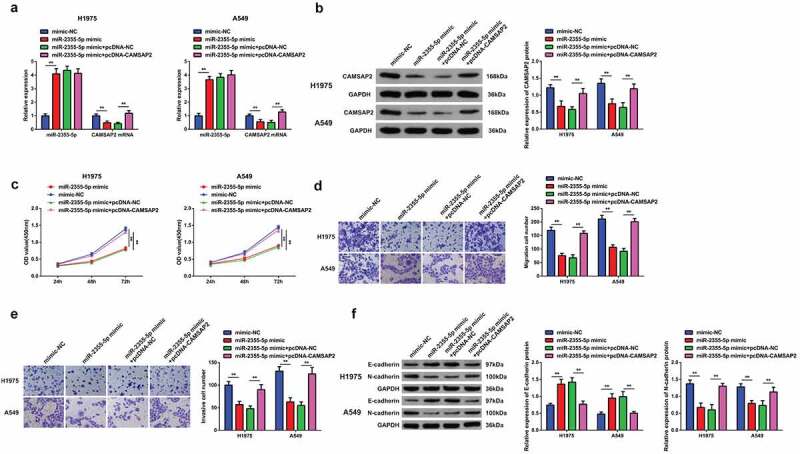
Elevated CAMSAP2 reverses the effects of strengthening miR-2355-5p on NSCLC cell advancement. A/B: RT-qPCR and Western blot to detect miR-2355-5p and circSOD2; C: CCK-8 to detect cell proliferation; D/E: Transwell to detect cell migration and invasion abilities; F: Western blot detection of E-cadherin and N-cadherin after cell transfection; *P < 0.05; **P < 0.01. N = 3. The data in the figure were manifested as mean ± SD.

In order to investigate whether miR-2355-5p had a regulatory impact on the progression of NSCLC cells and miR-2355-5p could target CAMSAP2, cells A549 and H1975 have been further transfected with elevated miR-2355-5p plasmid. We found that overexpression of miR-2355-5p downregulated the CAMSAP2, while over expression of pcDNA-CAMSAP2 successfully reversed the repression of elevated miR-2355-5p on CAMSAP2 (Figure 6a,b). Results have shown that over expression of miR-2355-5p, A549, and H1975 cells resulted in the reduced rate of tumor cell proliferation (Figure 6b-e), while pcDNA-CAMSAP2 turned around the changes induced via strengthening miR-2355-5p on NSCLC advancement. It assured that miR-2355-5p modulated the biological progress of NSCLC cells via targeting CAMSAP2.

## 4 Discussion

Lung cancer is a leading cause of cancer-linked deaths worldwide owing to its high frequency of incidence and ineffective late-stage treatment. Understanding its pathogenesis is necessary to provide a target for treatment [6]. CircRNAs are extensively expressed in neoplastic diseases and participate in the progression of a variety of tumors. A large number of studies have confirmed that circRNAs are linked with NSCLC, including numerous pathological processes, like proliferation, metastasis, autophagy, drug resistance, etc. [25–27]. In this study, we explored the characteristic mechanism of circSOD2 in NSCLC development. Results have shown that NSCLC cells and tissues present higher expression of circSOD2 as compared with normal tissues and cells. However, the process of NSCLC development can be reversed by knocking down the expression of CircSOD2, followed by upregulation of CAMSAP2 and miR-2355-5p. Previous studies suggested that mir-23355-5p provides basic characteristics of cancer such as sustained in cell proliferation and activated in metastasis, while suppressing its expression can lead to reversal of oncogenesis in lung adenocarcinoma [22].Our studies go in hand with the given study as CircSOD2 works as a ceRNA sponge to regulate miR-2355-5p expression and found that miR-2355-5p was downregulated and negatively linked with CircSOD2 in NSCLC cells and tissues. On the other hand, miR-2355-5p inhibition reverses the changes induced via refrained CircSOD2 on NSCLC advancement. CAMSAP2 was an immediate target of miR-2355-5p, and CAMSAP2 expression is higher in NSCLC cells and tissues. However, miR-2355-5p was negatively linked with CAMSAP2. Enhancive CAMSAP2 reversed the changes stimulated via elevated miR-2355-5p on NSCLC advancement.

In our study, we demonstrated that CircSOD2 exists as an oncogene in NSCLC advancement, and the main mechanism of its function is via performing as the sponge adsorbing miR-2355-5p, thereby controlling CAMSAP2. Similar to our study, Zhongwei Zhao *et al*. found that CircSOD2 is increased in HCC tumor tissues versus normal liver tissues. CircSOD2 acts as a sponge to adsorb miR-502-5p, represses miR-502-5p, and upregulates the expression of DNMT3a, which is target gene of miR-502-5p, thus regulating liver cancer cell progression and tumor growth *in vivo* [11]. We also find that CircSOD2 expression in NSCLC cells and tissues was augmented vs normal tissues and cells, indicating that CircSOD2 can be a potential biomarker for NSCLC diagnosis. This also echoes many previous studies in which circRNAs can be applied as biomarkers for the diagnosis of NSCLC and novel targets for treatment [28–30].

MiRNAs are single-stranded noncoding modulatory RNAs that repress protein expression via base pairing, thereby influencing the process of mRNA translation or degradation. Studies have shown that miR-2355-5p plays different functions in different cancers [31]. In the study, miR-2355-5p was reduced in NSCLC cells and tissues. MiR-2355-5p was negatively linked with CircSOD2 in NSCLC tissue. Repressive miR-2355-5p reversed the changes induced via refrained circSOD2 on NSCLC advancement. Elevated miR-2355-5p resists the occurrence and advancement of NSCLC. A similar trend was seen in the study by Chen D *et al*., where miR-2355-5p was reported to bind with DDX11-AS1 and refrain cell proliferation in breast cancer [32].

MiRNAs usually function as negative regulators of target genes, further manipulating tumor development and progression [19,33]. The investigation identified CAMSAP2 as a direct target of miR-2355-5p. CAMSAP2 is a part of the CAMSAP/Nezha/Patronin family. Studies have found that the CAMSAP/Patronin/Nezha family controls cell advancement with differentiation via modulating the negative dynamics of microtubules and noncentrosome microtubule assembly. Meanwhile, CAMSAP2 depletion impairs cell migration [34,35]. The research data supported that CAMSAP2 could bind to miR-2355-5p and augmented in NSCLC. Upregulation of CAMSAP2 could reverse the effect of miR-2355-5p on NSCLC progression. It assured that miR-2355-5p further modulated the onset and metastasis of NSCLC via targeting CAMSAP2.

In brief, in the study, it was found that CircSOD2 is elevated in tumors and cells of NSCLC patients and its silencing can partially curb NSCLC cell development through the CircSOD2/miR-2355-5p/CAMSAP2 ceRNA pathway, thereby refraining NSCLC malignant transformation. However, the research still has certain limitations. First, the results only described the in vitro studies, and no animal experiments have been carried out. This requires further research later. Second, cancer development is a very complicated process with multiple signaling pathway involvement. In our study, we only investigated the CircSOD2/miR-2355-5p/CAMSAP2 axis in NSCLC, while the downstream mechanism of CAMSAP2 on NSCLC has not been further studied. Dongxiao Li *et al*. believe that CAMSAP2 facilitates the migration of liver cancer cells via modulating noncentrosome microtubule acetylation modification. It is suspected that the effect of CAMSAP2 on NSCLC cells may also be a similar mechanism. This requires to be further enriched for signal pathway research in the future [20]. In general, the research has illuminated that the reduction of CircSOD2 upregulates miR-2355-5p to suppress the malignant behaviors of NSCLC cells via silencing CAMSAP2, thus decelerating the progression of NSCLC. These findings provide a new insight into a novel target therapy for NSCLC.

## Conclusion

It can be concluded that circSOD2 and CAMSAP2 are increased, but miR-2355-5p is decreased in NSCLC tumor tissues and cell lines. Meanwhile, CircSOD2 acts as a ceRNA to bind with miR-2355-5, and influences its activity of binding and negatively regulating the expression of CAMSAP2. Finally, the increased expression of CAMSAP2 reversed the changes stimulated by the elevated level of miR-2355-5p in NSCLC progression. This innovative signaling axis CircSOD2/miR-2355-5p/CAMSAP2 opens the new horizon to investigate NSCLC tumorigenesis in a better way.

## Highlights

1. Silencing or overexpression of CircSOD2 resulted in increased or decreased expression of miR-2355-5P, respectively.
2. circRNA works as sponge to absorb MiR-2355-5p and binds to the CircSOD2 and negatively regulates its expression.
3. CircSOD2 binds with miR-2355-5p, which was negatively regulating CAMSAP2 in NSCLC growth.
